# A Case Report on Pneumocephalus That Occurred Following an Epidural Ozone Injection During Percutaneous Lumbar Disc Decompression Surgery

**DOI:** 10.5812/aapm-142519

**Published:** 2024-03-07

**Authors:** Karim Hemati, Parnian Hemati, Saeid Rahimi Ghasabeh, Gholam Ali Dikafraz Shokooh

**Affiliations:** 1 Pain Research Center, Department of Anesthesiology and Pain Medicine, Iran University of Medical Sciences, Tehran, Iran; 2 Peoples’ Friendship University of Russia, Rudn University, Moscow, Russia

**Keywords:** Lumbar Decompression Surgery, Pneumocephalus

## Abstract

Spinal decompression is a common procedure in spinal, neurosurgery, and orthopedic surgery. While there are a number of known complications associated with it, pneumocephalus (air in the brain) is generally not a recognized complication postoperatively. However, in rare cases, it can occur as a result of spinal decompression surgery. We describe a case of a 54-year-old female patient who developed pneumocephalus following percutaneous lumbar disc decompression surgery of the lumbar spine. The patient presented to the emergency department 3 hours after discharge with severe restlessness, cognitive impairment, nausea, vomiting, and lack of balance. During symptomatic treatment in the emergency department and 1 hour after taking oxygen, the patient’s vital signs improved. Before discharge, a computed tomography (CT) scan was taken again, which showed the disappearance of radiological symptoms. The patient was discharged 12 hours after hospitalization with suitable clinical conditions. Obtaining urgent imaging tests (magnetic resonance imaging [MRI] or CT) at the cranial and spinal levels, along with an electroencephalogram, allows us to diagnose the problem and determine the appropriate course of treatment, whether pharmacological or surgical.

## 1. Introduction

Pneumocephalus is a condition in which gas, usually air, abnormally collects inside the skull (1). It was first observed in 1866 by Thomas, who examined a patient who had died from trauma (2). Pneumocephalus can have different causes; however, it mostly occurs after head injuries or brain surgeries (3). Pneumocephalus can occur when the skull is broken, from injuries to the head or face, tumors at the base of the skull, or after surgery or procedures on the brain, nose, and throat. It can also occur without any apparent cause. Tension pneumocephalus can be caused by a valve that allows air to enter the skull (4).

Pneumocephalus is very rare as a complication of surgeries on the spine, such as lumbar decompression for spinal problems (5). Most cases of pneumocephalus after surgery on the spine have been related to lumbar arthrodesis or similar surgeries that cause a hole in the dura mater (6).

In a series of 284 patients, symptoms included headaches (38%), nausea and vomiting, seizures, dizziness, and low neurological status (7). In clinical practice, it is important to distinguish between simple and tension pneumocephalus. The latter refers to a collection of air under higher pressure than the outside air pressure when a valve mechanism allows air to enter the skull but prevents it from leaving, resulting in a pressure difference (8). Spinal, neuro, and orthopedic surgeons often perform spinal decompression, along with intradiscal ozone, to treat acute or chronic degenerative spinal pathologies.

Although there are several recognized complications associated with spinal decompression surgery, pneumocephalus is not a common one of them (9). Here is a case report of pneumocephalus after decompression surgery of the lumbar spine.

## 2. Case Presentation

A 54-year-old female who has been experiencing lumbar radicular pain for the past 2 years did not respond to various treatments, including medications, rest, and physical therapy. In May 2023, she was referred to a private clinic in Tehran, Iran. The patient had no history of underlying diseases, except for controlled hypertension, and weighed 65 kg. Magnetic resonance imaging (MRI) results revealed swelling of the optic disc at L3-L4 and disc protrusions at L4-L5 and L1-L5. However, no anatomical issues or spondylolisthesis were detected in the spine.

Radiofrequency (RF) disc decompression and intradiscal ozone injection were recommended for the patient. The patient was fully monitored after being transferred to the outpatient operating room bed in the prone position. Under sterile conditions and guided by the fluoroscope, the right-sided disc space between L4 and L5 was identified, and the needle entry site was anesthetized with 3 cc of 2% lidocaine.

Then, using a #15 RF needle, the disc was accessed. After adjusting the needle location under fluoroscopy guidance and injecting 1 cc of water-soluble contrast agent, the appropriateness of the anteroposterior (AP) and lateral oblique positions was confirmed. The following procedures were then performed:

• Pulse Rf for 5 minutes (42 degrees Celsius)

• Lesion Rf for 1 minute (60 degrees Celsius)

• Lesion Rf for 1/5 minutes (90 degrees Celsius)

Subsequently, 6 cc of ozone (30 µ/cc) was injected into the inner space of the disk. The same procedures were also performed on discs L1-L5. Afterward, 3 cc of lidocaine was injected into the caudal epidural space in the same position under sterile conditions. Then, a G18 epidural needle was inserted into the site, and after confirming its position in anterior, posterior, and lateral views, injecting 2 cc of water-soluble contrast material, and observing a caudal epidural Christmas tree appearance, an epidural block was performed with Marcaine 25% + sodium hypertonic 5% 6 cc + triamcinolone 40 mg.

The epidural catheter was then advanced up to the L5-S1 level, and after re-checking its location, a mixture of Marcaine 25%, triamcinolone 40 mg, and sterile water up to 5 cc was injected. Finally, 5 cc of ozone (25 µ/cc) was injected through the epidural catheter. After finishing the work and dressing the area, the patient was transferred to the recovery room in good general condition and stable clinical condition. About half an hour after the patient was transferred to the recovery room, where patients are usually discharged, the case became very restless and complained of nausea and vomiting. The patient was given sedation and ondansetron 4 mg in two stages.

The patient’s symptoms decreased; however, she still experienced severe restlessness. Her vital signs and blood pressure were normal, and her blood oxygen saturation in room air was 95%. Her pupils and reaction to light were normal, and there was no neurological disorder.

**Figure 1.**
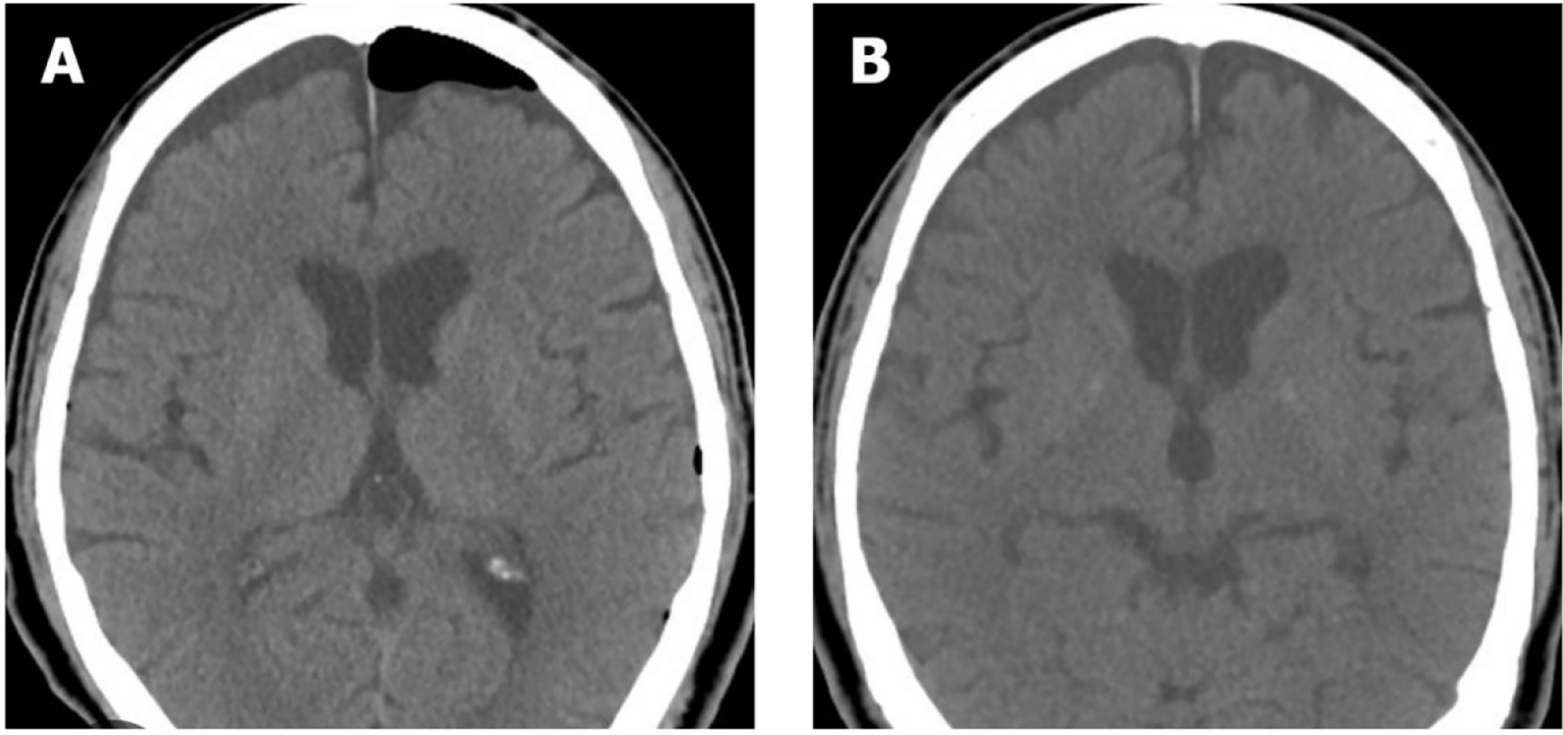
The brain computed tomography (CT) scan; A, before treatment; B, after treatment

According to the conditions, the patient was discharged one hour later, although she showed signs of restlessness. About 3 hours after discharge, her companions called and reported that she experienced severe restlessness, cognitive impairment, nausea, and vomiting. She lost her balance at home and fell from the bed. Due to her clinical condition and uncontrolled symptoms, she was taken to the hospital, where a brain computed tomography (CT) scan was performed without injection. Based on the results of this scan, she was diagnosed with pneumocephalus (Figure 1). 

The patient was admitted to the hospital and administered oxygen through a face mask at 5 - 10 liters per minute for 3 hours, followed by a nasal cannula. Her vital signs improved during symptomatic treatment in the emergency department and one hour after receiving oxygen. Before discharge, another CT scan was performed, which revealed no radiological abnormalities. The patient was discharged in stable clinical condition 12 hours after hospitalization.

## 3. Discussion

There are many causes of pneumocephalus, such as skull defects (e.g., transsphenoidal or endoscopic sinus surgery, craniotomy, and twist-drill drainage of chronic subdural hematomas), post-traumatic conditions (e.g., infection with gas-producing organisms, fractures through air sinuses or skull bases, neoplasms causing tumor erosion through the skull or skull base, osteomas, epidermoid, and pituitary tumors), and post-invasive procedures (e.g., Barotrauma, ventriculostomy, spinal anesthesia, and lumbar puncture) (10). Pneumocephalus might be a consequence of inadvertent dural perforation during procedures, such as spinal or epidural anesthesia. This can also be a result of employing the “loss of resistance to air” method in epidural blocks, which involves the injection of a minimal volume of air to discern the epidural space. Furthermore, an incidental dural puncture during a spinal or epidural procedure can precipitate this condition (11).

Depending on local practice patterns and specialties, practitioners commonly encounter a variety of etiologies associated with pneumocephalus. Craniotomy is one of the leading causes of pneumocephalus, and some cases are inevitable (12). According to Kim et al., 66% of postcraniotomy CT scans showed 5-10% oxygen in the intracranial volume on at least one axial CT section, and each postoperative scan showed at least trace oxygen (13). Although pneumocephalus is usually asymptomatic in most patients, it can cause postoperative lethargy, headaches, confusion, hemiparesis, and abducens nerve palsy when it reaches a sufficient volume (14).

Decompression of the lumbar spine can result in lumbar dural tears, cerebrospinal fluid (CSF) leakage, retroperitoneal inflammation, nerve root injury, pseudoarthrosis, infection, instability, and injury to the nerve roots. The procedure that causes pneumocephalus has not been established (15). The presence of a dural tear might function as a ball-valve mechanism through which air enters the cranial cavity through the foramen magnum. A negative pressure is created to draw air into and through the spinal canal and cranial cavity. However, air cannot escape from the spinal canal. As a result, both a defective dura and decreased intracranial pressure caused by CSF leakage contribute to the development of pneumocephalus (16).

Tension pneumocephalus is a condition in which excessive air is present in the brain, causing abnormal neurological signs and symptoms. As a result, the patient might experience rapid deterioration due to the appearance of an expanding lesion occupying the intracranial space. In addition to headaches, restlessness, disorientation, and confusion are other symptoms of this condition (17).

In this case, severe restlessness, cognitive impairment, nausea, vomiting, and lack of balance were observed 3 hours after discharge. According to Garcia-Garcia et al., a 76-year-old man was diagnosed with pneumocephalus after revision laminectomy was performed at L3 - L4 and L4 - L5 to decompress the lateral recesses at these levels. Although no obvious mass effect was observed on the initial postoperative CT scan of the patient, pneumocephalus might have been present in the recovery room immediately after initiating headache and confusion (18). According to Bonome Roel et al., a 54-year-old female patient developed pneumocephalus after spinal surgery. As a result of the large volume of intracranial gas in the patient, numerous complications can occur, including anesthesia-induced hypoxia, tonic-clonic seizures, and even status epilepticus (17).

Pneumocephalus associated with lumbar arthrodesis occurs when the dura mater is opened during surgery. Pneumocephalus might be caused by increased intra-abdominal pressure from decreased intra-abdominal pressure or by removal of the calcified disc, which could help facilitate intra-abdominal air entry. The entry of air into the subarachnoid space can be explained using the inverted bottle effect theory. Alternatively, the negative suction generated by the lumbar drain might facilitate the entry of intradural air and extraction of CSF. This theory could be valid when one drain tube is disconnected, allowing air to enter due to the negative pressure of the other drainage tube (19).

To prevent pneumocephalus during surgery, it is important to protect the dura mater. If injured, irrigating with saline can prevent air from entering, and surgically closing it can prevent the entry of air. Slightly positioning the patient in a Trendelenburg position can also help prevent air from entering the intradural compartment. Additionally, avoiding the use of nitrous oxide during anesthesia, which diffuses into air spaces, is an effective way to reduce the risk of pneumocephalus (20).

Tension pneumocephalus or indirect signs of intracranial hypertension might indicate an urgent need to evacuate a patient with pneumocephalus if the symptoms are minimal. Pneumocephalus is typically treated conservatively by avoiding elevating the head of the bed to more than 30°, providing hydration, analgesia, antiemetics, anticonvulsant prophylaxis, and monitoring the patient (5). In several case reports, supplemental oxygen has been shown to decrease intracranial air collection in patients with pneumocephalus (21). In a study involving 13 patients with postoperative pneumocephalus, those who breathed 100% oxygen using a nonrebreather mask for 24 hours showed a 65% reduction in pneumocephalus volume, compared to 31% for the control group (22).

The literature does not provide a standard treatment for pneumocephalus following surgery or skull base surgery. The relationship between pneumocephalus and a skull base defect is similar to the relationship between CSF leaks and postoperative pneumocephalus, suggesting similar treatment. Based on Viswanathan et al.'s findings, small bone defects causing pneumocephalus respond well to conservative measures, such as bed rest, head-of-bed elevation, and pain management (23).

Despite the absence of evidence of intracranial hypertension in our case and the progression of the patient’s symptoms, pneumocephalus was not evacuated because there was no evidence of respiratory distress in the patient. Within 12 hours, the patient no longer suffered from pneumocephalus and was no longer experiencing any symptoms. It is recommended that an electroencephalogram be performed to determine brain function and diagnose a decreased level of consciousness during surgery.

Ozone therapy is a potential treatment option for herniated discs that offers both pain relief and anti-inflammatory benefits. This therapy involves the use of a specialized machine that combines ozone with oxygen at a predetermined concentration. The mixture was then administered via a polypropylene syringe for a procedure known as discolysis, which is a non-surgical approach for managing disc prolapse (24). Ozone therapy has been observed to be generally safe, with a low rate of procedural complications, estimated to be around 0.1%. The side effects, while uncommon, can include a range of symptoms, such as insomnia, itching, the formation of small bumps around the injection site, gastritis, dizziness, rapid heart rate, and hot flashes (25).

Numerous studies have analyzed the effectiveness of ozone therapy, primarily in patients with herniated discs or those experiencing persistent symptoms even after surgical intervention, often referred to as failed back syndrome. The aforementioned studies suggest a success rate of approximately 75 - 80% for this treatment (26). Although generally considered safe, ozone therapy can occasionally lead to complications. These are rare but can include vitreoretinal hemorrhage and paresthesia associated with damage to the spinal nerves. Rao et al. reported a case of pneumocephalus in a 62-year-old female following an epidural injection of bupivacaine and ozone for the treatment of a prolapsed disc (27). Toman et al. demonstrated that the use of ozone and ozonated growth factors in the treatment of disc prolapse was carried out in 60 patients, with only two reported cases of pneumocephalus (28).

Although ozone therapy is generally considered safe, there have been isolated reports of unique complications by Vanni et al. (25). These complications include bilateral vitreoretinal hemorrhage, vertebrobasilar stroke, and paresthesia in the anterolateral portion of the left leg and foot. There have also been a few temporary episodes of impaired bilateral sensitivity, a case of L5 - S1 discitis, a single case of fatal septicemia, and the previously mentioned pneumocephalus. Long-term complications were observed 12 and 24 months after treatment. These are primarily characterized by the formation of tough adhesions between soft tissues and bones. In particular, severe adhesions of the nerve root to the dural sac have been reported (25).

In the case discussed here, the patient experienced severe and widespread headaches immediately after ozone treatment. Two potential mechanisms have been suggested for pneumocephalus development. The first involves leakage of CSF; however, the second involves intrathecal injection of air. It is important to note that severe headache can be a symptom of pneumocephalus, particularly when there is at least 2 mL of air in the subarachnoid area (29). In the present case, 5 mL of ozone was used. It is believed that ozone behaved similarly to air, leading to the sudden onset of a severe headache due to pneumocephalus.

Anesthesia complications can arise from various factors, such as airway manipulation, vascular access, patient positioning, and peripheral nerve blockade. These complications can include bronchospasm, laryngospasm, obstructive pulmonary edema, and even life-threatening anaphylaxis triggered by substances, such as neuromuscular-blocking agents, antibiotics, latex, hypnotic agents, opioids, and colloids. On the other hand, ozone therapy can lead to complications, such as inflammation in various organs, irritation if ozone enters the mouth, nose, or eyes, and serious conditions, such as spine infections, septicemia, and abscesses. (30).

It is noteworthy that the influence of anesthesia on lung inflammation induced by ozone is not extensively documented. For instance, the administration of isoflurane prior to exposure to ozone has the potential to suppress inflammatory reactions in the lungs. Nevertheless, the pre-exposure administration of a combination of ketamine, xylazine, and atipamezole can exacerbate both pulmonary and systemic inflammation. Consequently, the selection of an anesthetic can have a substantial effect on a patient’s reaction to ozone therapy (31). The interaction between anesthesia and ozone therapy can also result in unique complications, making a comprehensive understanding of these factors crucial for patient care.

### 3.1. Conclusions

Ozone therapy is a safe and cost-effective method for treating patients with back pain who have not responded to conservative therapy. However, in some instances, this treatment can lead to side effects, such as pneumocephalus. Although pneumocephalus is a rare condition, it can occur after spinal surgery due to anesthesia. In most cases, there are no symptoms associated with pneumocephalus. However, it can cause headaches, confusion, nausea, vomiting, seizures, dizziness, and/or focal neurological symptoms, such as hemiparesis and/or cranial nerve palsies. Experiencing headaches after undergoing ozone treatment, a common therapy for lower back pain, should be taken seriously. In such cases, it is crucial to consider a differential diagnosis of pneumocephalus. Obtaining urgent imaging tests (MRI or CT) at the cranial and spinal levels, along with an electroencephalogram, allows us to diagnose the problem and determine the appropriate course of treatment, whether pharmacological or surgical.

## Data Availability

The dataset presented in the study is available on request from the corresponding author during submission or after publication.

## References

[1] Álvarez-Holzapfel MJ, Aibar Durán JÁ, Brió Sanagustin S, de Quintana-Schmidt C (2019). [Diffuse pneumocephalus after lumbar stab wound].. An Pediatr (Engl Ed)..

[2] Thomas A (1865). Du pneumatocele du crane..

[3] Cunqueiro A, Scheinfeld MH (2018). Causes of pneumocephalus and when to be concerned about it.. Emerg Radiol..

[4] McCarthy CJ, Behravesh S, Naidu SG, Oklu R (2017). Air Embolism: Diagnosis, Clinical Management and Outcomes.. Diagnostics (Basel)..

[5] Gauthe R, Latrobe C, Damade C, Foulongne E, Roussignol X, Ould-Slimane M (2016). Symptomatic compressive pneumocephalus following lumbar decompression surgery.. Orthop Traumatol Surg Res..

[6] Wang JC, Tsai SH, Liao WI (2014). Pneumocephalus after epidural anesthesia in an adult who has undergone lumbar laminectomy.. J Neurosurg Anesthesiol..

[7] Barr DL, McDonald BS (2021). Iatrogenic pneumocephalus following a cervical epidural steroid injection: A case report.. Radiol Case Rep..

[8] Abu-Hamdiyah OJ, Al Sharie S, Awadi S, Khamees A, Athamneh MJ (2021). Pneumocephalus secondary to a spinal surgery: A literature review and a case report.. Int J Surg Case Rep..

[9] Lim Y, Dahapute A, Clarke A, Hutton M, Selbi W (2023). Delayed tension pneumocephalus and pneumorrhacis after routine cervical spine surgery treated successfully without burr holes.. Ann R Coll Surg Engl..

[10] Kim BJH, Ji MY, Chen JCC, Correia JA, Law AJJ, Kow CY (2024). Use of oxygen therapy for pneumocephalus: a systematic review.. Neurosurg Rev..

[11] Koo J, Cho KT (2020). Pneumocephalus and Chemical Meningitis after Inadvertent Dural Puncture during Lumbar Epidural Injection.. Korean J Neurotrauma..

[12] Jumah A, Alsaif A, Fana M, Aboul Nour H, Zoghoul S, Eltous L (2024). Spinal procedures, pneumocephalus, and cranial nerve palsies: A review of the literature.. Neuroradiol J..

[13] Kim TK, Yoon JR, Kim YS, Choi Y, Han S, Jung J (2022). Pneumocephalus and headache following craniotomy during the immediate postoperative period.. BMC Surg..

[14] Porras JL, Rowan NR, Mukherjee D (2022). Endoscopic Endonasal Skull Base Surgery Complication Avoidance: A Contemporary Review.. Brain Sci..

[15] Aassouani F, Ennacery Z, Bensalah A, Charifi Y, Mamadou D, El Bouardi N (2022). Lumbar puncture as a cause of tension pneumocephalus, pneumorrachis, and sacral meningocele infection leading to death: An extremely rare case report.. Radiol Case Rep..

[16] Gao H, Ma HJ, Li YJ, Yin C, Li Z (2020). Prevalence and risk factors of postoperative delirium after spinal surgery: a meta-analysis.. J Orthop Surg Res..

[17] Bonome Roel C, Goday Etxebarria M, Domenech Bendana C, Montero Picallo A, Vieira Lopez BI (2023). Pneumocephalus, coma and seizures following lumbar decompression surgery.. Rev Esp Anestesiol Reanim (Engl Ed)..

[18] Garcia-Garcia D, Gomez-Rice A, Vázquez-Vecilla I, López-Franco M (2021). Tension Pneumocephalus: a Case Report of a Rare Complication After Spinal Surgery.. SN Comprehensive Clinical Medicine..

[19] Yang C, Chiu C, Wu C (2022). Diffuse symptomatic pneumocephalus after biportal endoscopic spinal surgery: illustrative case.. J Neurosurg: Case Lessons..

[20] Szymaszek M, Toshkezi G (2020). Mount Fuji sign following nasal polypectomy: Conservative management of pneumocephalus.. Interdiscip Neurosurg..

[21] Li W, Liu Q, Lu H, Wang H, Zhang H, Hu L (2020). Tension Pneumocephalus from Endoscopic Endonasal Surgery: A Case Series and Literature Review.. Ther Clin Risk Manag..

[22] Begum F, Moningi S, Murthy TN (2023). High-Flow Nasal Oxygen Therapy for Management of Postoperative Pneumocephalus.. J Neuroanaesth Crit Care..

[23] Viswanathan R, Sanjeevi V, Dhandapani B (2020). Unusual and Rare Pneumocephalus Presentations in a Tertiary Care Center: Management Strategies and Review of Literature.. Indian J Neurosurg..

[24] Daghedy M, Rabie A, Elwany A, Moussa W (2023). Percutaneous Pulsed Radiofrequency versus Combined Intradiscal Oxygen-Ozone Therapy with Percutaneous Radiofrequency for Management of Discogenic Cervical Radiculopathy.. Pan Arab J Neurosurg..

[25] Vanni D, Galzio R, Kazakova A, Pantalone A, Sparvieri A, Salini V (2015). Intraforaminal ozone therapy and particular side effects: preliminary results and early warning.. Acta Neurochirurgica..

[26] Sconza C, Leonardi G, Kon E, Respizzi S, Massazza G, Marcacci M (2021). Oxygen-ozone therapy for the treatment of low back pain: a systematic review of randomized controlled trials.. Eur Rev Med Pharmacol Sci..

[27] Rao SM, Kotgire L, Sastri BVS (2019). Pneumocephalus Caused by an Epidural Ozone Injection for Treatment of Disc Prolapse.. Res Pract Anesthesiol Open J..

[28] Toman H, Ozdemir U, Kiraz HA, Luleci N (2017). Severe headache following ozone therapy: Pneumocephalus.. Agri..

[29] Ahmad M, Bellamy S, Ott W, Mekhail R (2023). Pneumocephalus secondary to epidural analgesia: a case report.. J Med Case Rep..

[30] Hidalgo-Tallon FJ, Torres-Morera LM, Baeza-Noci J, Carrillo-Izquierdo MD, Pinto-Bonilla R (2022). Updated Review on Ozone Therapy in Pain Medicine.. Front Physiol..

[31] Wilson ML, Thysell JA, Baumann KK, Quaranta DV, Liang WS, Erickson MA (2022). Effects of Anesthesia on Ozone-Induced Lung and Systemic Inflammation.. Lung..

